# Prepulse Inhibition of the Startle Reflex as a Predictor of Vulnerability to Develop Locomotor Sensitization to Cocaine

**DOI:** 10.3389/fnbeh.2019.00296

**Published:** 2020-02-03

**Authors:** M. Carmen Arenas, María Carmen Blanco-Gandía, José Miñarro, Carmen Manzanedo

**Affiliations:** ^1^Unidad de investigación Psicobiología de las Drogodependencias, Departamento de Psicobiología, Facultad de Psicología, Universitat de València, Valencia, Spain; ^2^Departamento de Psicología y Sociología, Facultad de Ciencias Sociales y Humanas, Universidad de Zaragoza, Campus de Teruel, Teruel, Spain

**Keywords:** prepulse inhibition, cocaine, male and female mice, endophenotype, behavioral sensitization, motor effects, biomarker

## Abstract

Prepulse inhibition (PPI) of the startle reflex is a measure of sensory-motor synchronization. A deficit in PPI has been observed in psychiatric patients, especially those with schizophrenia and vulnerable subjects, since the neural bases of this disorder are also involved in the regulation of PPI. Recently, we have reported that baseline PPI levels in mice can predict their sensitivity to the conditioned reinforcing effects of cocaine in the conditioned place preference (CPP) paradigm. Mice with a low PPI presented a lower sensitivity to the conditioned rewarding effects of cocaine; however, once they acquired conditioned preference with a higher dose of the drug, a more persistent associative effect of cocaine with respect to environmental cues was evident in these animals when compared with High-PPI mice. Therefore, we proposed that the PPI paradigm can determine subjects with a higher vulnerability to the effects of cocaine. Developing locomotor sensitization after pre-exposure to cocaine is considered an indicator of transitioning from recreational use to a compulsive consumption of the drug. Thus, the aim of the present study was to evaluate whether subjects with a low PPI display a higher locomotor sensitization induced by cocaine. First, male and female OF1 mice were classified as High- or Low-PPI according to their baseline PPI levels. Subsequently, the motor effects induced by an acute dose of cocaine (Experiments 1 and 2) and the development of locomotor sensitization induced by pre-exposure to this drug (Experiments 3 and 4) were recorded using two apparatuses (Ethovision and actimeter). Low-PPI mice presented low sensitivity to the motor effects of an acute dose of cocaine, but a high increase of activity after repeated administration of the drug, thus suggesting a great developed behavioral sensitization. Differences after pretreatment with cocaine vs. saline were more pronounced among Low-PPI subjects than among High-PPI animals. These results endorse our hypothesis that the PPI paradigm can detect subjects who are more likely to display behaviors induced by cocaine and which can increase the risk of developing a cocaine use disorder. Herein, we further discuss whether a PPI deficit can be considered an endophenotype for cocaine use disorder.

## Introduction

Cocaine use is a serious health problem with important social and economic consequences. Cocaine is the most widely consumed illegal stimulant drug in Europe and North America, mainly by the young adult population [European Monitoring Centre for Drugs and Drug Addiction (EMCDDA, 2019)]. It is one of the drugs with the fastest transition from abuse to compulsive use (Flórez-Salamanca et al., 2013), and its addiction presents a persistent and high susceptibility to relapse (Volkow et al., 2016). Additionally, its compulsive use is often associated with multiple cardiovascular, neurological, and psychiatric disorders (Galicia et al., 2014; González-Llona et al., 2015; Kariisa et al., 2019). Unfortunately, there is as of yet no approved treatment deemed effective for dependence on this drug (Czoty et al., 2016; Volkow and Boyle, 2018). In this context, obtaining physiological markers, as endophenotypes, that identify the individuals that are most vulnerable to developing a cocaine use disorder is a priority for research in this area. An endophenotype is considered a biomarker of genetic vulnerability that can be measured reliably and indicates a possible risk of a psychiatric disorder with clinical state independency (Beauchaine, 2009). Moreover, it must meet certain criteria, such as distinction from illness in the general population, inheritability, and presence at a higher rate in non-affected family members than in the general population (Gottesman and Gould, 2003).

Deficiencies in the inhibitory mechanisms of the brain, of dopaminergic origin primarily, can result in low levels of self-control in individuals, related in turn to a greater predisposition to developing compulsive drug use (Belin et al., 2013). The prepulse inhibition (PPI) of the startle response is a neurophysiological measure of sensory-motor gating. A deficit in PPI may indicate alterations in the cerebral dopaminergic system; for example, in the mesolimbic pathway (Swerdlow et al., 2016). This deficit is considered an endophenotype of schizophrenia, and has been observed in many other psychiatric disorders (Swerdlow and Light, 2016).

Recently, given that neural structures regulating PPI and drug addiction have been shown to coincide (Volkow and Morales, 2015; Arenas et al., 2017), we have begun to evaluate the predictive ability of PPI in order to identify animals that are more sensitive to the effects of cocaine. In a previous study (Arenas et al., 2018), we showed that the baseline PPI level of mice predicts their sensitivity to the conditioned reinforcing effects of cocaine in the conditioned place preference (CPP) paradigm. Mice with a high PPI—both males and females—acquired CPP with a subthreshold dose of cocaine (1 mg/kg) and with an effective dose (6 mg/kg), while animals with a low PPI did not present conditioned preference with either of these two doses. Males and females with a lower PPI required higher doses of cocaine (12 mg/kg) to acquire conditioned preference than those with a high PPI; in other words, they were less sensitive to the conditioned reinforcing effects of the drug. Nevertheless, among the mice with a low PPI, it took longer to extinguish the conditioned preference in males, while preference was reinstated with lower doses than High-PPI animals in females. These behavioral differences could have been related to differences in the levels of D1 and D2 dopamine receptors in the striatum of these animals. For instance, mice with a low PPI, especially females, presented higher levels of D2 receptor expression (Arenas et al., 2018).

The increased motor response to a drug after repeated exposure is a phenomenon termed behavioral sensitization or motor sensitization (for review, see Steketee and Kalivas, 2011). Development of this sensitization is a possible indicator of the probability of transitioning from recreational use to a compulsive consumption of drugs (Robinson and Berridge, 2003, 2008) and a behavioral representation of drug-induced synaptic plasticity (Lüscher and Malenka, 2011; Steketee and Kalivas, 2011). Long-term sensitization may underlie drug craving and relapse to cocaine use (Steketee, 2005). It has recently been demonstrated that intermittent access to a drug is more effective in producing long-term changes in the brain and addiction-like behaviors, with intermittent patterns of use being especially pronounced during the transition to addiction, prior to regular use (Kawa et al., 2019). This neuroplasticity induced by cocaine use is observed specifically in the dopaminergic system, from the ventral tegmental area to the nucleus accumbens (NAc), which is also a central circuit for the regulation of PPI (Doherty et al., 2008; Rohleder et al., 2016). Moreover, the behavioral effects of cocaine are mediated by D1 and D2 receptors, which have also been associated with baseline PPI levels (Doherty et al., 2008; Arenas et al., 2018). If a PPI deficit indicates alterations in the mesolimbic dopaminergic system (Swerdlow et al., 2016), it is possible that a subject with low PPI is more vulnerable to the cerebral changes induced by cocaine use.

Furthermore, the aim of the present study was to evaluate whether PPI can also be considered an endophenotype for vulnerability to developing sensitization to cocaine-induced motor effects in male and female mice. For this purpose, male and female OF1 mice were classified as High- or Low-PPI according to their baseline PPI levels. The motor effects induced by an acute dose of cocaine (10 mg/kg) and the development of motor sensitization induced by pre-exposure to this drug (Ferrer-Pérez et al., 2018) were then recorded. There are many procedures that can be performed to measure the motor activity of mice. In our laboratory, we primarily use two automatic methods to record the activity of animals in an open field: a computerized video-tracking system named Ethovision and an apparatus with infrared lights, namely, an actimeter (Blanco-Gandía et al., 2017; Ferrer-Pérez et al., 2018; García-Pardo et al., 2019). These two automatic procedures for measuring the motor activity of mice assess the different aspects of the motor response; the Ethovision program measures the distance traveled in cm by the animal, while the actimeter records both the horizontal and vertical activity of the mice. According to previous experience, we hypothesized that the motor response of male and female mice induced by cocaine would not be the same in the two procedures, since they each evaluate different aspects of the motor response. Many authors suggest employing a battery of different tests that measure the same behavioral parameters in order to obtain more reliable information about the behavior in question (Kalueff et al., 2007; Ramos, 2008). Given that our objective was to demonstrate the predictive capacity of the PPI paradigm to detect vulnerability to developing motor sensitization regardless of the procedure used, we decided to use both methods. Additionally, we considered it necessary to evaluate the motor effect of an acute dose of cocaine (10 mg/kg), as we expected that Low-PPI mice would display lower sensibility to the effects of cocaine than High-PPI mice, in accordance with the results in Arenas et al. (2018). Although mice received a single dose of cocaine in the sensitization procedure, those in the control group had previously received a regime of saline injections, which could have created a stressful context that increased the sensibility of mice to the effects of cocaine (Ferrer-Pérez et al., 2018, 2019).

## Materials and Methods

### Subjects

A total of 253 (131 males and 122 females) mice of the OF1 strain (Charles River, Barcelona, Spain) were employed in the four experiments. The animals arrived at the laboratory at 6 weeks of age and were all housed in groups of four in plastic cages (28 cm length × 28 cm width × 14.5 cm height) under the following conditions according to RD 1201/2005: constant temperature (21 ± 2°C), a relative humidity of 60%, an inverted 12-h light cycle (white lights on 19:30–7:30), and food and water available ad libitum (except during behavioral tests).

Procedures involving mice and their care were conducted in conformity with national, regional, and local laws and regulations, which are in accordance with the Directive 2010/63/EU of the European Parliament and of the council of September 22, 2010, on the protection of animals used for scientific purposes. The Animal Use and Care Committee of the University of Valencia approved the present study: 2014/VSC/PEA/00118 and 2016/VSC/PEA/00132.

### Drugs

Cocaine hydrochloride (Laboratorios Alcaliber, Spain) was diluted in physiological saline (0.9% NaCl) at a volume of 0.01 ml/g and injected intraperitoneally (IP) at a dose of 10 mg/kg for locomotor activity and 25 mg/kg to produce sensitization. Control mice were injected IP with the corresponding volume of physiological saline.

### Apparatus

#### Prepulse Inhibition (PPI)

Two PPI devices were used, each consisting of a Plexiglas tube (28 ± 15 ± 17 cm) on top of a platform with a sensor at its base that measures the force applied by the animal to the platform after a sound stimulus. The value used in the study was the peak value of the startle response. This value was translated by an accelerometer and the signal was collected and digitized by a computer. The apparatus (mod startle response CERS) and the software were purchased from CIBERTEC, S.A, Madrid. Spain. The description of the unit is found in Arenas et al. (2018).

#### Open Field Recorded by Ethovision

The apparatus was composed of four Plexiglas open-field chambers (30 cm long ± 30 cm wide ± 35 cm high) in which locomotor activity was registered by a computerized video-tracking system (Ethovision, Noldus S.A., The Netherlands). The movements of the mouse inside the open-field chambers were recorded and translated automatically by the software to horizontal distance traveled (in cm) for every 10-min period.

#### Open Field Recorded by Actimeter

Locomotor activity was measured automatically by an actimeter (CIBERTEC S.A., Spain) consisting of eight cages (33 × 15 × 13 cm), each with eight infrared lights located in a frame around the cage (see Mateos-García et al., 2015). In this apparatus, beams were positioned on the horizontal axis 2 cm apart, at a height just above the bottom of the cage (body level of the mice). The different frames were separated from each other at a distance of 4 cm and, since they were opaque, prevented the animals from seeing conspecifics.

### Procedures

In separate studies, after categorization according to PPI, the animals were evaluated with the Ethovision apparatus (Experiment 1) and actimeter (Experiment 2) to measure the motor response induced by 10 mg/kg of cocaine. The locomotor sensitization induced by 25 mg/kg of cocaine was also measured using Ethovision (Experiment 3) and the actimeter (Experiment 4). Locomotor activity tests were performed when mice had entered adulthood (Experiment 1: PND 71–88; Experiment 2: PND 55–74; Experiment 3: PND 58–75; Experiment 4: PND 68–70).

#### PPI Procedure

After an adaptation period of 5 days, all the mice about to be included in an experiment performed the pre-pulse inhibition test (PPI), on PND 46–65. The procedure, similar to that used in previous studies (Arenas et al., 2018), was carried out in two phases (acclimation and test) over 2 days. On the first day, mice were placed in the animal holder for 5 min with a constant 65-dB white noise as background noise, but without startle stimuli. On the second day, the PPI test was performed. This phase also began with 5 min of 65-dB white noise, but was followed by a program of stimuli in which the white noise continued to be present. The program consisted of two phases: the first was a series of 50 trials of 120-dB pulses to establish the baseline—this pulse was chosen as it was the maximum value reached in the startle response in pilot studies—and the PPI was evaluated in the second. In order to obtain a more stable value of the PPI level, two different prepulse intensities were employed (75 and 85 dB during 4 ms each), along with two different inter-stimulus intervals (30 ms and 100 ms) and one single pulse at an intensity of 120 dB, for 20 ms each. Thus, four types of prepulse-pulse trials were performed—75 dB/30 ms, 75 dB/100 ms, 85 dB/30 ms and 85 dB/100 ms—all of them followed by a 120-dB pulse. To determine the value of the PPI, we ran the four types of prepulse-pulse trials alongside single instances of the 75, 85, and 120 dB tones, 10 times each, in pseudorandom order, giving a total of 70 trials separated by a 20-s interval. The prepulses (75/85 dB) were introduced to verify that they were not acting as pulses and that the 120-dB pulse was the only stimulus inducing the startle response in the animal. The total duration of phase 2 was 45 min.

PPI was calculated as a percentage score: PPI (%) = 100 − (startle response for pulse with pre-pulse ± 100/startle response for pulse alone), and the mean of the four PPI obtained (75 dB/30 ms, 75 dB/100 ms, 85 dB/30 ms, and 85 dB/100 ms) was used to divide the animals into high or low PPI by means of a two-cluster analysis of K mean. Cluster centers were determined separately by sex to allow for equal distribution of males and females in each PPI group. Mice (10 males and eight females) with a PPI level near the cutoff point between High- and Low-PPI were removed from the experiment to emphasize the difference between High- and Low-PPI animals, and to avoid fluctuation of the cut off point among the samples in each Experiment, which can lead to errors. The final experimental groups are shown in Table 1.

**Table 1:** Classification as High-Prepulse inhibition (PPI) or Low-PPI mice using a two-cluster analysis (mean ± SEM).

		Low-PPI		High-PPI	
		Mean ± SEM	*n*	Mean ± SEM	*n*
Experiment 1	Males	4.05 ± 3.5	10	34.6 ± 1.9	11
	Females	5.5 ± 3.2	9	36.8 ± 1.9	10
Experiment 2	Males	2.5 ± 2.5	9	24.6 ± 2.6	10
	Females	2.8 ± 2.3	9	28.1 ± 2.9	10
Experiment 3	Males saline	9 ± 2.9	10	35.3 ± 2.9	12
	Females saline	−4.8 ± 3.4	9	25.9 ± 2.3	9
	Males cocaine	8.3 ± 3.7	9	33.3 ± 1.9	11
	Females cocaine	−6.8 ± 5	9	26.8 ± 2.1	9
Experiment 4	Males saline	−4.1 ± 4.3	10	29.5 ± 3.5	10
	Females saline	−12.1 ± 4.1	8	36.5 ± 2.6	12
	Males cocaine	−6.8 ± 3.8	9	30.3 ± 3.5	10
	Females cocaine	−8.9 ± 4.7	8	37.2 ± 3.7	12

*Percentage of PPI (mean of the four PPI obtained at 75 dB/30 ms, 75 dB/100 ms, 85 dB/30 ms, and 85 dB/100 ms) was used to classify the animals according to their PPI levels. A two-cluster analysis of K means was performed for the distribution of the animals within each sex according to their higher/lower PPI level*.

#### Cocaine-Induced Behavioral Sensitization

The protocol for motor sensitization induced by cocaine involved three phases: the sensitization induction phase, in which three administrations of physiological saline or cocaine 25 mg/kg were performed on consecutive days, once per day; the sensitization development phase, an interval of 5 days during which no injections were administered; and the test phase, in which locomotor activity induced by 10 mg/kg of cocaine (challenger) was evaluated. This procedure was selected based on previous reports showing that it evokes locomotor sensitization induced by cocaine in mice (Ferrer-Pérez et al., 2018).

To assess the locomotor response or locomotor sensitization induced by cocaine, we recorded the first 60 min before administering the drug (phase of habituation to the environment) and the 30 min after (phase of test). The activity data were grouped in 10-min intervals. During the 90-min recording period, males and females were not kept together in the same room.

### Statistical Analysis

Statistical analyses were performed using the IBM SPSS Statistics v24.0 software (Systat Software Inc., Chicago, IL, USA). In order to obtain a more stable value of PPI level, a mean of the four PPI percentage obtained (75 dB/30 ms, 75 dB/100 ms, 85 dB/30 ms and 85 dB/100 ms) was used to classify the animals according to their PPI levels. A cluster analysis of K means was performed for the distribution of the animals within each sex according to their higher/lower PPI level. A one-way ANOVA with a dependent variable (PPI) was performed in each experiment to check whether there were differences in the PPI between sexes. For the locomotor activity data, a mixed ANOVA was performed with two between variables—Sex (males and females) and PPI (High and Low)—and one within variable, Minutes (six 10-min periods to analyze locomotor activity in the habituation phase, and four 10-min periods to analyze locomotor activity in the test phase). For the behavioral sensitization data, a mixed ANOVA was performed with three between variables—Sex (males and females), PPI (High and Low) and Pre-Treatment (Saline and Cocaine)—and one within variable, Minutes (six 10-min periods to analyze locomotor activity in the habituation phase, and four 10-min periods to analyze locomotor activity in the test phase), using Bonferroni’s pairwise *post hoc* comparisons. In order to test our hypothesis about varying vulnerability to developing behavioral sensitization in Low- and High-PPI mice, we also performed the Tukey’s honest significant difference (HSD) test, which does not require previous ANOVA with a significant interaction between factors and is highly conservative against type I error (Wilcox and Rousselet, 2018). All results are expressed as mean ± SEM. Differences were considered statistically significant when *p* < 0.05.

## Results

### Experiment 1: Locomotor Activity Induced by Cocaine (10 mg/kg) Recorded by Ethovision

**Figure 1:**
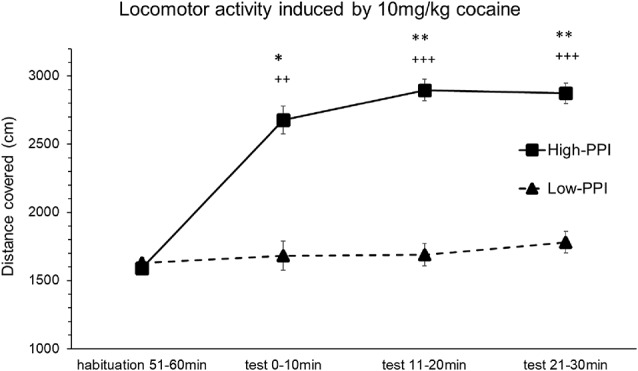
Locomotor response induced by an acute dose of cocaine (10 mg/kg) recorded by Ethovision. Locomotor response induced by 10 mg/kg of cocaine in mice (both males and females) categorized as high and low prepulse inhibition (PPI). Data presented as mean values ± SEM during the 30-min period of distance covered in centimeter. *p < 0.05, **p < 0.01 Low-PPI vs. High-PPI; ^++^*p* < 0.01, ^+++^*p* < 0.001 vs. habituation.

Male and female mice were classified as High-PPI or Low-PPI animals using a two-cluster analysis (*F*_(1,21)_ = 62.552; *p* < 0.0001, in males; *F*_(1,19)_ = 71.962; *p* < 0.0001, in females). The ANOVA performed for the data obtained during the whole period (60 min) revealed an effect of habituation to the environment (*F*_(5,32)_ = 33.841; *p* < 0.0001), showing that motor activity in all the animals decreased after the first 10 min until 60 min (*p*s < 0.01). This analysis also revealed a significant effect of the variable Sex (*F*_(1,36)_ = 13.387; *p* < 0.001), with females presenting higher locomotor activity than males (data not shown). The results of the motor activity of male and female mice together in response to 10 mg/kg of cocaine are presented in Figure 1. The ANOVA showed a significant effect of the variable Minutes (*F*_(3,34)_ = 8.006; *p* < 0.0001), as all animals together significantly increased their activity at 20 min (*p* < 0.002) and at 30 min (*p* < 0.0001), and displayed a tendency towards an increase at 10 min (*p* = 0.056). A significant effect of the variable PPI (*F*_(1,36)_ = 8.148; *p* < 0.007) and the interaction Minutes*PPI (*F*_(3,34)_ = 5.487; *p* < 0.003) was also observed. The animals with a high PPI displayed a higher locomotor activity than Low-PPI mice: High-PPI mice increased their locomotor activity at 10 min (*p* < 0.005), at 20 min (*p* < 0.0001), and at 30 min (*p* < 0.0001) with respect to habituation. High-PPI mice also exhibited more activity than those with a low PPI at 10 min (*p* < 0.041), at 20 min (*p* < 0.001), and at 30 min (*p* < 0.002). The interaction Sex*Minutes*PPI was not significant.

### Experiment 2: Locomotor Activity Induced by Cocaine (10 mg/kg) Recorded by Actimeter

**Figure 2:**
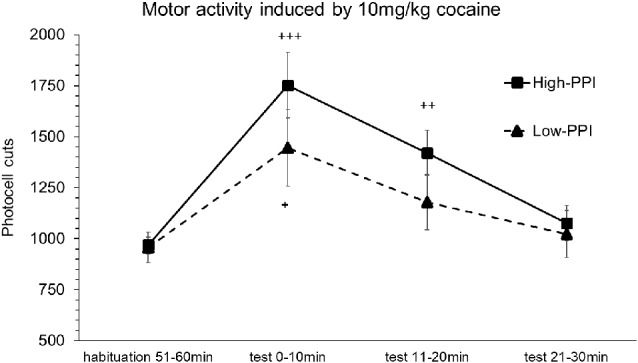
Motor response induced by an acute dose of cocaine (10 mg/kg) recorded by actimeter. Motor response induced by 10 mg/kg of cocaine in mice (both males and females) categorized as High- and Low-PPI. Data presented as mean values ± SEM of photocell cuts during the 30 min period. ^+^*p* < 0.05, ^++^*p* < 0.01, ^+++^*p* < 0.001 vs. habituation.

Male and female mice were classified as High-PPI or Low-PPI animals using a two-cluster analysis: male (*F*_(1,17)_ = 43.313; *p* < 0.0001) and female (*F*_(1,17)_ = 42.490; *p* < 0.0001). The ANOVA performed for the data obtained during the whole period (60 min) revealed an effect of habituation to the environment (*F*_(5,30)_ = 70.451; *p* < 0.0001), demonstrating that all the animals decreased their motor activity after the first 10 min until 60 min (*p*s < 0.01). This analysis also revealed a significant effect of the variable Sex (*F*_(1,34)_ = 14.825; *p* < 0.0001), with males presenting a higher locomotor activity than females (data not shown). The results of motor activity induced by 10 mg/kg of cocaine are presented in Figure 2. The analysis revealed a significant effect of the variable Minutes (*F*_(3,32)_ = 9.977; *p* < 0.0001), with all the animals displaying higher activity with respect to habituation at 10 min (*p* < 0.0001) and at 20 min (*p* < 0.001). The interactions Minutes*PPI and Sex*Minutes*PPI were not significant. However, the Tukey HSD test indicated that cocaine induced hyperactivity at 10 min (*p* < 0.042) in all Low-PPI mice, and at 10 min (*p* < 0.0001) and 20 min (*p* < 0.001) in all High-PPI mice.

### Experiment 3: Locomotor Sensitization Induced by Cocaine Recorded by Ethovision

**Figure 3:**
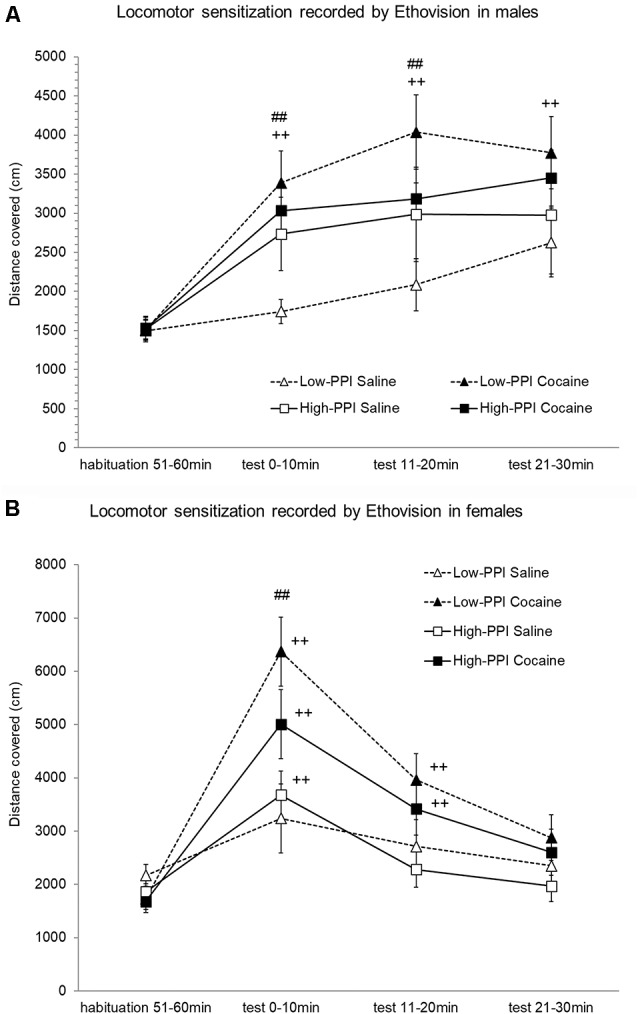
**(A)** Cocaine sensitization recorded by Ethovision in males. During the induction phase, the animals received a pre-treatment with saline or 25 mg/kg cocaine per day on three consecutive days. Five days later, all the animals received a 10 mg/kg challenge. Data presented as mean values ± SEM during the 30-min period of distance covered in centimeter. ^##^*p* < 0.01 Low-PPI cocaine vs. saline; ^++^*p* < 0.01 Low-PPI cocaine, High-PPI cocaine and saline vs. habituation. **(B)** Cocaine sensitization recorded by Ethovision in females. During the induction phase, the animals received a pre-treatment with saline or 25 mg/kg cocaine per day on three consecutive days. Five days later, all the animals received a 10 mg/kg challenge. Data presented as mean values ± SEM during the 30-min period of distance covered in centimeter. ^##^*p* < 0.01 Low-PPI cocaine vs. saline; ^++^*p* < 0.01 vs. habituation.

Male and female mice were classified as High-PPI or Low-PPI animals using a two-cluster analysis: male (*F*_(1,43)_ = 75.968; *p* < 0.0001) and female (*F*_(1,36)_ = 67.667; *p* < 0.0001). The ANOVA performed for the data obtained during the whole period (60 min) revealed an effect of habituation to the environment (*F*_(5,66)_ = 39.709; *p* < 0.0001), with a decrease in motor activity after the first 10 min until 60 min observed in all animals (data not shown). However, the interaction Minutes*Sex was significant (*F*_(5,66)_ = 3.487; *p* < 0.007), with males displaying higher activity in the first 10 min (*p* < 0.04) and lower activity in the last 60 min (*p* < 0.04) in comparison with females. The results of motor activity in response to 10 mg/kg of cocaine (the challenger) after pre-treatment with physiological saline or cocaine 25 mg/kg are presented in Figures 3A,B. The analysis revealed a significant effect of the variable Minutes (*F*_(3,68)_ = 53.674; *p* < 0.0001), with all animals displaying higher activity induced by the cocaine challenge for all time points with respect to the habituation phase (*p*s < 0.001), and a higher activity to the first 10 min than 20 min (*p* < 0.02) and 30 min (*p* < 0.05). The variable Pre-Treatment (*F*_(1,70)_ = 14.394; *p* < 0.0001) and the interaction Minutes*Pre-Treatment (*F*_(3,68)_ = 10.323; *p* < 0.0001) were significant, revealing higher activity in mice pretreated with cocaine than those pretreated with saline to the first 10 min (*p* < 0.0001), 20 min (*p* < 0.001), and 30 min (*p* < 0.05). The interaction Minutes*PPI*Pre-Treatment almost reached significance (*F*_(3,68)_ = 2.262; *p* < 0.08). The Tukey HSD test indicated higher hyperactivity induced by the cocaine challenge in mice pretreated with cocaine with respect to those pretreated with saline only in Low-PPI mice—females at 10 min (*p* < 0.0001) and males at 10 min (*p* < 0.029) and at 20 min (*p* < 0.004)—with no significant differences observed between the High-PPI mice pretreated with cocaine vs. saline. Moreover, with respect to the habituation phase, a significant increase of activity was observed in Low-PPI males pretreated with cocaine at 10 min (*p* < 0.002), 20 min (*p* < 0.0001), and 30 min (*p* < 0.0001); in High-PPI males pretreated with saline at 10 min (*p* < 0.037), 20 min (*p* < 0.004), and 30 min (*p* < 0.01); in High-PPI males pretreated with cocaine at 10 min (*p* < 0.008), 20 min (*p* < 0.001), and 30 min (*p* < 0.001); in Low-PPI females pretreated with cocaine at 10 min (*p* < 0.0001) and 20 min (*p* < 0.0001); in High-PPI females pretreated with saline at 10 min (*p* < 0.003); and in High-PPI females pretreated with cocaine at 10 min (*p* < 0.0001) and 20 min (*p* < 0.003).

### Experiment 4: Locomotor Sensitization Induced by Cocaine Recorded by Actimeter

**Figure 4:**
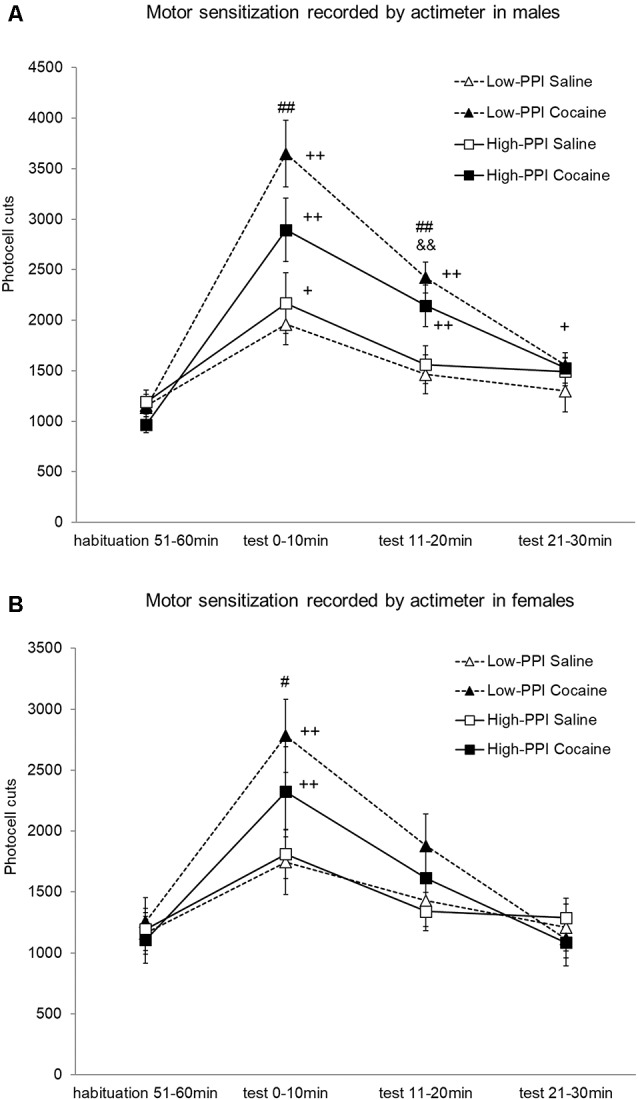
**(A)** Cocaine sensitization recorded by the actimeter in males. During the induction phase, the animals received a pre-treatment with saline or 25 mg/kg cocaine per day on three consecutive days. Five days later, all the animals received a 10 mg/kg challenge. Data presented as mean values ± SEM of photocell cuts during the 30-min period. ^##^*p* < 0.01 Low-PPI cocaine vs. saline; ^&&^*p* < 0.01 High-PPI cocaine vs. saline; ^+^*p* < 0.05, ^++^*p* < 0.01 vs. habituation. **(B)** Cocaine sensitization recorded by the actimeter in females. During the induction phase, the animals received a pre-treatment with saline or 25 mg/kg cocaine per day on three consecutive days. Five days later, all the animals received a 10 mg/kg challenge. Data presented as mean values ± SEM of photocell cuts during the 30-min period. ^#^*p* < 0.05 Low-PPI cocaine vs. saline; ^++^*p* < 0.01 vs. habituation.

Male and female mice were classified as High-PPI or Low-PPI using a two-cluster analysis: male (*F*_(1,42)_ = 79.646; *p* < 0.0001) and female (*F*_(1,42)_ = 102.673; *p* < 0.0001; see Figure 4A). The ANOVA for the data obtained during the whole period (60 min) revealed an effect of habituation to the environment (*F*_(5,67)_ = 35.594; *p* < 0.0001), with all the animals decreasing their motor activity after the first 10 min until 60 min (data not shown). The results of motor activity in response to 10 mg/kg of cocaine challenge after pre-treatment with physiological saline or 25 mg/kg of cocaine are presented in Figures 4A,B. The analysis revealed a significant effect of the variable Sex (*F*_(1,71)_ = 5.306; *p* < 0.024), with higher activity in males than in females; the variable Pre-Treatment (*F*_(1,71)_ = 10.527; *p* < 0.002), with higher activity among the mice pretreated with cocaine vs. saline; and the variable Minutes (*F*_(3,69)_ = 46.105; *p* < 0.0001), as the cocaine challenge increased activity with respect to the habituation phase in all the animals together at all the time points measured (*p*s < 0.001). Furthermore, the interaction Sex*Minutes (*F*_(3,69)_ = 3.939; *p* < 0.012) was significant, with males displaying more hyperactivity induced by cocaine than females at 10 min (*p* < 0.023), 20 min (*p* < 0.022), and 30 min (*p* < 0.016). The interaction Minutes*Pre-Treatment (*F*_(3,69)_ = 11.301; *p* < 0.0001) was also significant; mice pretreated with saline showed a significant increase of activity at 10 min (*p* < 0.0001), while those pre-treated with cocaine showed a significant increase of activity at 10 min (*p* < 0.0001) and 20 min (*p* < 0.0001) in comparison to the habituation phase. In this way, the mice pre-treated with cocaine exhibited higher hyperactivity than those pre-treated with saline at 10 min (*p* < 0.0001) and 20 min (*p* < 0.0001). No other significant interactions were observed. However, the Tukey HSD test indicated higher hyperactivity induced by the cocaine challenge in mice pretreated with cocaine vs. saline: in High-PPI males at 20 min (*p* < 0.041), in Low-PPI males at 10 min (*p* < 0.0001) and 20 min (*p* < 0.001), and in Low-PPI females at 10 min (*p* < 0.032). Moreover, with respect to the habituation phase, a significant increase of activity was observed in High-PPI males pretreated with saline at 10 min (*p* < 0.01); in High-PPI males pretreated with cocaine at 10 min (*p* < 0.0001), 20 min (*p* < 0.0001), and 30 min (*p* < 0.009); in Low-PPI males pretreated with cocaine at 10 min (*p* < 0.0001) and 20 min (*p* < 0.0001); in High-PPI females pretreated with cocaine at 10 min (*p* < 0.0001); and in Low-PPI females pretreated with cocaine at 10 min (*p* < 0.0001).

## Discussion

The main objective of the present study was to evaluate the predictive capacity of the PPI paradigm to identify subjects among male and female mice that are more vulnerable to the motor effects of cocaine and the development of behavioral sensitization induced by the drug. The results reveal for the first time that male and female mice with a low PPI display lower hyperactivity when administered an acute dose of cocaine (10 mg/kg), but that they seem to develop a higher motor sensitization to the drug after intermittent exposure. Low-PPI mice pretreated with cocaine presented more hyperactivity induced by cocaine than those pretreated with saline, while High-PPI animals were less affected by pretreatment with cocaine. In a previous study, we provided the first evidence that both male and female mice with a low PPI are more vulnerable to the conditioned rewarding and reinstating effects of cocaine (Arenas et al., 2018). We observed how Low-PPI mice displayed less sensitivity to the conditioned rewarding effects of cocaine, but a stronger and more persistent conditioned preference when conditioned with a higher dose. Therefore, we consider that Low-PPI mice seem to be more vulnerable to the rewarding and motor effects of cocaine.

In accordance with previous studies in male rodents (Abizaid et al., 2011; Blanco-Gandía et al., 2017), a dose of 10 mg/kg of cocaine induced hyperactivity in all mice together; however, we have now observed this motor effect of cocaine in both male and female mice when measured with two different procedures. Nonetheless, this dose did not increase the motor activity of animals with a low PPI in the open field when measured with the Ethovision and actimeter apparatuses. This lower sensibility to the motor effects of an effective dose of cocaine in Low-PPI mice is in line with the lower sensitivity of these subjects to the rewarding effects of drug with respect to High-PPI mice seen in our previous study (Arenas et al., 2018).

Furthermore, as expected, all the groups that received pretreatment with cocaine developed cocaine motor sensitization, as reported in other studies using the same (Ferrer-Pérez et al., 2018; García-Pardo et al., 2019) and other (Gracia-Rubio et al., 2016; Luján et al., 2018) experimental protocols. Thus, when all the mice pretreated with cocaine were compared with those pretreated with saline, it was evident that the drug had induced higher hyperactivity. Nonetheless, when subjects were separated into High- and Low-PPI groups, higher hyperactivity was induced by cocaine principally in latter, in both males and females, suggesting a higher motor sensitization in mice with a lower PPI, a result obtained with both Ethovision and actimeter protocols. Other authors have also reported a higher behavioral sensitization to the effects of other DA agonists, such as amphetamine, in Low-PPI vs. High-PPI mice, suggesting that males characterized by low basal PPI are more susceptible to the development of dopamine sensitization (Peleg-Raibstein et al., 2013). Motor sensitization, also called behavioral sensitization, is the increased motor activity induced by cocaine after intermittent drug administration (Steketee and Kalivas, 2011). It is considered an indicator of neural changes caused by cocaine consumption (Lüscher and Malenka, 2011), which can induce compulsive consumption of abuse drugs, a characteristic behavior of addiction (Robinson and Berridge, 2003, 2008). Intermittent patterns of use are especially pronounced during the transition to addiction, prior to regular use. Recently, it has been demonstrated that intermittent access to drugs is more effective than continued use in producing long-term changes in the brain and addiction-like behaviors (Kawa et al., 2019). The results of the present study highlight more pronounced behavioral changes after pretreatment with cocaine in mice with a lower PPI than in those with a higher PPI, a result that was once again observed in both males and females. In this way, Low-PPI mice pretreated with the drug presented higher hyperactivity when they received a cocaine challenge than when they were pretreated with saline, an increase of motor activity that was less marked among High-PPI mice pretreated with cocaine vs. saline. The marked behavioral sensitization of Low-PPI mice in the motor response induced by cocaine is also in accordance with results obtained previously in our laboratory (Arenas et al., 2018) regarding the reward response of these mice to cocaine. In the study in question, animals with low PPI also presented more pronounced addictive-like behavior in the CPP induced by cocaine than High-PPI mice, since the former mice required a higher dose of cocaine to acquire CPP (12 mg/kg). Once they were conditioned, preference was not extinguished in males and was reinstated in females with lower doses of cocaine than in their control counterparts (Arenas et al., 2018). It is known that both male and female rats develop addiction-like behaviors with intermittent administration experience, but that this occurs more rapidly and more robustly in females (Kawa et al., 2019). This greater effect in females is due to the “telescoping effect” described in the clinical literature, consisting of an accelerated progression from the initiation of substance use to the onset of dependence (Griffin et al., 1989; Kosten et al., 1993; Greenfield et al., 2010). Hence, our results seem to support the idea that alterations in the brain that underlie psychomotor sensitization also underlie sensitization to the incentive motivational effects of drugs (Robinson and Berridge, 1993; De Vries et al., 1998; Lorrain et al., 2000; Allain et al., 2017).

As a whole, these results suggest that the long-term consequences of use of psychostimulants such as cocaine or amphetamine can differ depending on basal levels of PPI. Other studies have reported that amphetamine has varying effects depending on the basal PPI level of subjects (Swerdlow et al., 2003; Talledo et al., 2009). In this context, amphetamine was shown to largely decrease PPI in subjects with the highest basal levels of PPI, in both men (Swerdlow et al., 2003) and women (Talledo et al., 2009). Other DA agonists, such as pergolide and amantadine, have been shown to reduce PPI in men with basal PPI above the median of the normal distribution, whereas they slightly amplified PPI in men with basal levels below the median (Bitsios et al., 2005). These seemingly opposite effects could be explained by the role of DA in the NAc in the modulation of PPI (Doherty et al., 2008; Rohleder et al., 2016). Amphetamine decreases PPI in a strain of rats with a relatively low COMT gene expression in the NAc, while it increases PPI in another strain of rats with relatively high COMT gene expression in the NAc (Talledo et al., 2009). In light of this evidence, DA availability in the NAc seems to be critical in determining the level of PPI observed; i.e., a higher DA level will induce a PPI deficit, while a lower DA level will increase PPI (Arenas et al., 2017). The role of DA in determining PPI levels has been bolstered since activation of midbrain cholinergic neurons has been shown not to mediate PPI levels (Azzopardi et al., 2018). One of the main brain nuclei that modulate PPI is the pedunculopontine tegmentum (PPTg; Arenas et al., 2017), which is one of the major cholinergic centers of the brain (Gut and Winn, 2016). However, it has recently been demonstrated that cholinergic PPTg neurons do not mediate PPI, in contrast to a longstanding hypothesis maintaining that they do (Azzopardi et al., 2018). Furthermore, the lack of motor and rewarding effects of cocaine observed in the mice with a low PPI level may reflect an altered DA system. Hence, these animals, especially females, show higher levels of D2R expression than those with a higher PPI level (Arenas et al., 2018), and high D2 receptor levels in the striatum have been related to a lack of pleasant effects perceived from psychostimulants (Volkow et al., 1999). Indeed, clinical studies have reported that schizophrenic patients display higher amounts of D2 receptors (Seeman, 2013) in addition to a PPI deficit (Braff, 2010). Some individuals at familial risk of addictions exhibit higher striatal D2R availability (Volkow et al., 2006), although it is still under debate whether these elevated D2R densities are protective against or a risk for addiction (Leyton, 2017).

We also observed sex differences in the spontaneous activity of mice in the habituation sessions. In general, it is considered that female rodents are more active than males (Blizard et al., 1975), though this difference depends on the age, environment, and procedure used (Tamás et al., 2005; Alstott and Timberlake, 2009; Bogdanova et al., 2013). In the present study, females presented a higher locomotor activity when measured with the Ethovision apparatus, while males showed a higher motor activity when measured with the actimeter. These differences between males and females indicate that the procedures for evaluating animal activity measure different behaviors. In this context, the Ethovision protocol for measuring the motor activity of mice records ambulatory activity; in other words, it provides the distance in cm traveled by the animal. Our female animals displayed a higher ambulatory activity in this protocol, in line with a previous study with a different strain of mice (Sershen et al., 2002) and another with rats (Blizard et al., 1975). In contrast, the actimeter apparatus recorded all the motor activity of the animal, since it did not distinguish locomotion from stereotypic or grooming movements, with males showing a higher activity than females in this case.

In summary, the results of our study are in accordance with those of our previous study (Arenas et al., 2018), and endorse the predictive capacity of the PPI paradigm to identify individuals who are more likely to display behaviors induced by cocaine and which can increase the risk of developing a cocaine use disorder. Bearing all the discussed results in mind, subjects with a lower PPI present a cocaine response that entails a higher risk of transitioning from abuse to dependence. First, they present lower sensitivity to the motor and reinforcing effects of cocaine, which may drive them to higher consumption rates. Second, when Low PPI mice are exposed to higher doses of cocaine, they present stronger associative cocaine effects with environmental cues than High PPI mice, which leads to a more persistent drug-seeking behavior. Third, mice with a lower PPI exposed to intermittent administration of cocaine present more pronounced behavioral changes; that is, they develop great behavioral sensitization induced by the drug, which is considered an indicator of the possible transition to a compulsive consumption of drugs, a characteristic behavior of addiction (Robinson and Berridge, 2003, 2008; Lüscher and Malenka, 2011). Furthermore, we demonstrate this enhanced motor sensitization induced by cocaine using two different protocols for evaluating motor activity. It is important to point out that a deficit in PPI has been observed in many psychiatric disorders characterized by alterations in the cerebral dopaminergic system, and is thus already considered an endophenotype of schizophrenia (Swerdlow and Light, 2016). Additionally, the dopaminergic system, from the ventral tegmental area to the NAc, is considered a central circuitry for the regulation of PPI (Doherty et al., 2008; Rohleder et al., 2016) and motivation for reward and drug-seeking (Bergamini et al., 2016; Scofield et al., 2016). Therefore, we endorse PPI paradigm as a useful indicator of subjects with a higher vulnerability to the effects of cocaine. Notwithstanding, future studies should be performed to confirm whether a deficit in PPI is a possible endophenotype of cocaine use disorder.

## Data Availability Statement

The datasets generated for this study are available on request to the corresponding author.

## Ethics Statement

The animal study was reviewed and approved by The Animal Use and Care Committee of the University of Valencia.

## Author Contributions

MA, MB-G, JM, and CM contributed to the conception and design of the study. MB-G and CM performed the experiments and collected and analyzed the data. MA and CM performed the statistical analyses. All authors interpreted the data. MA and MB-G wrote the manuscript.

## Conflict of Interest

The authors declare that the research was conducted in the absence of any commercial or financial relationships that could be construed as a potential conflict of interest.
